# Neighborhood Regularized Logistic Matrix Factorization for Drug-Target Interaction Prediction

**DOI:** 10.1371/journal.pcbi.1004760

**Published:** 2016-02-12

**Authors:** Yong Liu, Min Wu, Chunyan Miao, Peilin Zhao, Xiao-Li Li

**Affiliations:** 1 Joint NTU-UBC Research Centre of Excellence in Active Living for the Elderly (LILY), and School of Computer Engineering, Nanyang Technological University, Singapore; 2 Institute for Infocomm Research (I^2^R), Agency for Science, Technology and Research (A*STAR), Singapore; National Center for Biotechnology Information (NCBI), UNITED STATES

## Abstract

In pharmaceutical sciences, a crucial step of the drug discovery process is the identification of drug-target interactions. However, only a small portion of the drug-target interactions have been experimentally validated, as the experimental validation is laborious and costly. To improve the drug discovery efficiency, there is a great need for the development of accurate computational approaches that can predict potential drug-target interactions to direct the experimental verification. In this paper, we propose a novel drug-target interaction prediction algorithm, namely neighborhood regularized logistic matrix factorization (NRLMF). Specifically, the proposed NRLMF method focuses on modeling the probability that a drug would interact with a target by logistic matrix factorization, where the properties of drugs and targets are represented by drug-specific and target-specific latent vectors, respectively. Moreover, NRLMF assigns higher importance levels to positive observations (i.e., the observed interacting drug-target pairs) than negative observations (i.e., the unknown pairs). Because the positive observations are already experimentally verified, they are usually more trustworthy. Furthermore, the local structure of the drug-target interaction data has also been exploited via neighborhood regularization to achieve better prediction accuracy. We conducted extensive experiments over four benchmark datasets, and NRLMF demonstrated its effectiveness compared with five state-of-the-art approaches.

## Author Summary

This work introduces a computational approach, namely neighborhood regularized logistic matrix factorization (NRLMF), to predicting potential interactions between drugs and targets. The novelty of NRLMF lies in integrating logistic matrix factorization with neighborhood regularization for drug-target interaction prediction. In NRLMF, we model the interaction probability for each drug-target pair using logistic matrix factorization. As the observed interacting drug-target pairs are experimentally verified, they are more trustworthy than the unknown pairs. We propose to assign higher importance levels to interaction pairs and lower importance levels to unknown pairs. In addition, we further improve the prediction accuracy by neighborhood regularization, which considers the neighborhood influences from most similar drugs and most similar targets. To evaluate the performance of NRLMF, we conducted extensive experiments on four benchmark datasets. The experimental results demonstrated that NRLMF usually outperformed five state-of-the-art methods under three different cross-validation settings, in terms of the area under the ROC curve (AUC) and the area under the precision-recall curve (AUPR). In addition, we confirmed the practical prediction ability of NRLMF by mapping with the latest version of four online biological databases, including ChEMBL, DrugBank, KEGG, and Matador.

## Introduction

The drug discovery is one of the primary objectives of the pharmaceutical sciences, which is an interdisciplinary research field of fundamental sciences covering biology, chemistry, physics, statistics, etc. In the drug discovery process, the prediction of drug-target interactions (DTIs) is an important step that aims to identify potential new drugs or new targets for existing drugs. Therefore, it can help guide the experimental validation and reduce costs. In recent years, the DTI prediction has attracted vast research attentions and numerous algorithms have been proposed [1]. Existing methods predict DTIs based on a small number of experimentally validated interactions in existing databases, such as ChEMBL [2], DrugBank [3], KEGG DRUG [4], and SuperTarget [5]. Previous studies have shown that a fraction of new interactions between drugs and targets can be predicted based on the experimentally validated DTIs, and the computational methods for identifying DTIs can significantly improve the drug discovery efficiency.

In general, traditional computational methods proposed for DTI prediction can be categorized into two main groups: docking simulation approaches and ligand-based approaches [6–8]. The docking simulation approaches predict potential DTIs, considering the structural information of target proteins. However, the docking simulation is extensively time-consuming, and the structural information may not be available for some protein families, for example the G-protein coupled receptors (GPCRs). In the ligand-based approaches, potential DTIs are predicted by comparing a candidate ligand with the known ligands of the target proteins. This kind of approaches may not perform well for the targets with a small number of ligands.

Recently, the quick development of machine learning techniques provides effective and efficient ways to predict DTIs. An intuitive idea is to formulate the DTI prediction as a binary classification problem, where the drug-target pairs are treated as instances, and the chemical structures of drugs and the amino acid subsequences of targets are treated as features. Then, classical classification methods can be used, e.g., support vector machines (SVM) [9] and regularized least square (RLS) [10]. For example, in [11], a SVM model was utilized to classify a given drug-target pair into interaction and non-interaction, considering the amino acid sequences of proteins, chemical structures, and the mass spectrometry data. Bleakley and Yamanishi proposed a supervised approach for DTI prediction based on the bipartite local models (BLMs), where SVM was used to build the local models [12]. Xia et al. proposed a semi-supervised DTI prediction approach, namely Laplacian regularized least square (LapRLS), and extended it to incorporate the kernel constructed from the known DTI network [13]. van Laarhoven et al. defined a Gaussian interaction profile (GIP) kernel to represent the interactions between drugs and targets, and they employed RLS with the GIP kernel for DTI prediction problems [14, 15]. Cheng et al. developed three supervised inference methods for DTI prediction based on the complex network theory [16]. Mei et al. integrated BLM method with a neighbor-based interaction-profile inferring (NII) procedure to form a DTI prediction approach called BLM-NII, where the RLS classifier with GIP kernel was used as the local model [17]. Moreover, Yamanishi et al. developed a web server called DINIES, which utilized supervised machine learning techniques, e.g., pairwise kernel learning and distance metric learning, to predict unknown DTIs from different sources of biological data [18]. Ding et al. used a uniform experimental setting to empirically review the advantages and limitations of existing similarity-based learning approaches for DTI prediction [19]. Furthermore, other auxiliary information has also been exploited for DTI prediction. For example, in [20], Li et al. developed a computational framework that integrated literature mining and the protein and drug connectivity information derived from protein interaction networks to build the disease-specific drug-protein connectivity maps. In [21], Chen et al. utilized the data from public datasets to build a semantic linked network connecting drugs and targets. A statistical model was also proposed to evaluate the association of drug-target pairs.

Essentially, the DTI prediction problem is a recommendation task that aims to suggest a list of potential DTIs. Thus, another line of research for DTI prediction is the application of recommendation technologies. In the literature, collaborative filtering (CF) based approaches are the most widely adopted recommendation methods, which can be categorized into two main groups, i.e., memory-based CF and model-based CF approaches [22, 23]. As the most successful model-based CF approach, matrix factorization has been explored for DTI prediction in recent studies. For example, Gönen proposed a kernelized Bayesian matrix factorization (KBMF) method, which combined the kernel-based dimensionality reduction, matrix factorization, and binary classification for DTI prediction [24]. Cobanoglu et al. utilized probabilistic matrix factorization (PMF) [25] to predict unknown DTIs [26]. The accuracy of the PMF based approach was further improved by an active learning strategy. Moreover, Zheng et al. introduced the multiple similarities collaborative matrix factorization (MSCMF) model, which exploited multiple kinds of drug similarities and target similarities to improve the DTI prediction accuracy [27].

In this paper, we propose a novel matrix factorization approach, namely neighborhood regularized logistic matrix factorization (NRLMF), for DTI prediction. The proposed NRLMF method focuses on predicting the probability that a drug would interact with a target. Specifically, the properties of a drug and a target are represented by two latent vectors in the shared low dimensional latent space, respectively. For each drug-target pair, the interaction probability is modeled by a logistic function of the drug-specific and target-specific latent vectors. This is different from the KBMF method [24] that predicts the interaction probability using a standard normal cumulative distribution function of the drug-specific and target-specific latent vectors [28]. In NRLMF, an observed interacting drug-target pair (i.e., positive observation) is treated as *c* (*c* ≥ 1) positive examples, while an unknown pair (i.e., negative observation) is treated as a single negative example. As such, NRLMF assigns higher importance levels to positive observations than negatives. Because the positive observations are biologically validated and thus usually more trustworthy. However, the negative observations could contain potential DTIs and are thus unreliable. This differs from previous matrix factorization based DTI prediction methods [24, 26, 27] that treat the interaction and unknown pairs equally.

Additionally, NRLMF also studies the local structure of the interaction data to further improve the DTI prediction accuracy, by exploiting the neighborhood influences from most similar drugs and most similar targets. In particular, NRLMF imposes individual regularization constraints between the latent representations of a drug and its nearest neighbors, which are most similar with the given drug. Similar neighborhood regularization constraints have also been added on the latent representations of targets. Note that this neighborhood regularization method is different from previous approaches that exploit the drug similarities and target similarities using kernels [13, 14, 17, 29] or factorizing the similarity matrices [27]. Moreover, the proposed approach only considers nearest neighbors instead of all similar neighbors as used in previous approaches, avoiding noisy information, thus achieves more accurate results.

The performances of NRLMF were empirically evaluated on four benchmark datasets, compared with five state-of-the-art DTI prediction methods. Experimental results showed that NRLMF usually outperformed other competing methods on all datasets under different experimental settings, in terms of the widely adopted measures, i.e., the area under the ROC curve (AUC) and the area under the precision-recall curve (AUPR). In addition, the practical prediction ability of NRLMF was also confirmed by mapping with the latest version of online biological databases, including ChEMBL [2], DrugBank [30], KEGG [4], and Matador [5].

## Materials and Methods

### Materials

The performances of DTI prediction algorithms were evaluated on four benchmark datasets, including Nuclear Receptors, G-Protein Coupled Receptors (GPCR), Ion Channels, and Enzymes. These datasets were originally provided by [31] and were publicly available at http://web.kuicr.kyoto-u.ac.jp/supp/yoshi/drugtarget/. Table 1 summarizes the statistics of all four datasets. Each dataset contains three types of information: 1) the observed DTIs, 2) the drug similarities, and 3) the target similarities. Particularly, the observed DTIs were retrieved from public databases KEGG BRITE [32], BRENDA [33], SuperTarget [5], and DrugBank [3]. The drug similarities were computed based on the chemical structures of the compounds derived from the DRUG and COMPOUND sections in the KEGG LIGAND database [32]. For a pair of compounds, the similarity between their chemical structures was measured by the SIMCOMP algorithm [34]. The target similarities, on the other hand, were calculated based on the amino acid sequences of target proteins, which were retrieved from the KEGG GENES database [32]. The normalized Smith-Waterman score was used to compute the sequence similarity between two proteins.

**Table 1:** The statistics of the DTI datasets from [31].

	Nuclear Receptor	GPCR	Ion Channel	Enzyme
Number of drugs	54	223	210	445
Number of targets	26	95	204	664
Number of interaction pairs	90	635	1476	2926
Average number of drugs per target	3.46	6.68	7.24	4.41
Average number of targets per drug	1.67	2.85	7.03	6.58
Sparsity of the interaction matrix	93.59%	97.00%	96.55%	99.01%
Percentage of drugs that have only one interaction target	72.22%	47.53%	38.57%	39.78%
Percentage of targets that have only one interaction drug	30.77%	35.79%	11.27%	43.37%

### Problem Formalization

In this paper, the set of drugs is denoted by $D={\left\{d_{i}\right\}}_{i=1}^{m}$, and the set of targets is denoted by $T={\left\{t_{j}\right\}}_{j=1}^{n}$, where *m* and *n* are the number of drugs and number of targets, respectively. The interactions between drugs and targets are represented by a binary matrix $Y\in R^{m\times n}$, where each element *y*_*ij*_ ∈ {0, 1}. If a drug *d*_*i*_ has been experimentally verified to interact with a target *t*_*j*_, *y*_*ij*_ is set to 1; otherwise, *y*_*ij*_ is set to 0. The non-zero elements in **Y** are called “interaction pairs” and regarded as positive observations. The zero elements in **Y** are called “unknown pairs” and regarded as negative observations. We define the set of positive drugs and targets as $D^{+}=\left\{d_{i}|\sum_{j=1}^{n}y_{ij}>0,\forall 1\leq i\leq m\right\}$ and $T^{+}=\left\{t_{j}|\sum_{i=1}^{m}y_{ij}>0,\forall 1\leq j\leq n\right\}$, respectively. Then, the set of negative drugs (i.e., new drugs without any known interaction targets) and negative targets (i.e., new targets without any known interaction drugs) are defined as *D*^−^ = *D*∖*D*^+^ and *T*^−^ = *T*∖*T*^+^, respectively. In addition, the drug similarities are represented by $S^{d}\in R^{m\times m}$, where the (*i*, *μ*) element $s_{i\mu }^{d}$ is the similarity between *d*_*i*_ and *d*_*μ*_. The target similarities are described using $S^{t}\in R^{n\times n}$, where the (*j*, *ν*) element $s_{j\nu }^{t}$ is the similarity between *t*_*j*_ and *t*_*ν*_.

The objective of this study is to first predict the interaction probability of a drug-target pair and subsequently rank the candidate drug-target pairs according to the predicted probabilities in descending order, such that the top-ranked pairs are the most likely to interact.

### Logistic Matrix Factorization

The matrix factorization technique has been successfully applied for DTI prediction in previous studies. In this work, we develop the DTI prediction model based on logistic matrix factorization (LMF) [35], which has been demonstrated to be effective for personalized recommendations. The primary idea of applying LMF for DTI prediction is to model the probability that a drug would interact with a target. In particular, both drugs and targets are mapped into a shared latent space, with a low dimensionality *r*, where *r* ≪ min(*m*, *n*). The properties of a drug *d*_*i*_ and a target *t*_*j*_ are described by two latent vectors $u_{i}\in R^{1\times r}$ and $v_{j}\in R^{1\times r}$, respectively. Then, the interaction probability *p*_*ij*_ of a drug-target pair (*d*_*i*_, *t*_*j*_) is modeled by the following logistic function:

$$\begin{array}{c} p_{ij}=\frac{\exp(u_{i}v_{j}^{⊤})}{1+\exp(u_{i}v_{j}^{⊤})}. \end{array}$$ (1)

For simplicity, we further denote the latent vectors of all drugs and all targets by $U\in R^{m\times r}$ and $V\in R^{n\times r}$ respectively, where **u**_*i*_ is the *i*^*th*^ row in **U** and **v**_*j*_ is the *j*^*th*^ row in **V**.

In DTI prediction tasks, the observed interacting drug-target pairs have been experimentally verified, thus they are more trustworthy and important than the unknown pairs. Towards a more accurate modeling for DTI prediction, we propose to assign higher importance levels to the interaction pairs than unknown pairs. In particular, each interaction pair is treated as *c* (*c* ≥ 1) positive training examples, and each unknown pair is treated as a single negative training example. Here, *c* is a constant used to control the importance levels of observed interactions and is empirically set to 5 in the experiments. This importance weighting strategy has been demonstrated to be effective for personalized recommendations [35–37]. However, to the best of our knowledge, it has not been explored for DTI prediction in previous studies.

By assuming that all the training examples are independent, the probability of the observations is as follows:

$$\begin{array}{c} p\left(Y|U,V\right)=\left(\prod_{1\leq i\leq m,1\leq j\leq n,y_{ij}=1}{\left[p_{ij}^{y_{ij}}{\left(1-p_{ij}\right)}^{\left(1-y_{ij}\right)}\right]}^{c}\right)\times \\ \left(\prod_{1\leq i\leq m,1\leq j\leq n,y_{ij}=0}p_{ij}^{y_{ij}}{\left(1-p_{ij}\right)}^{\left(1-y_{ij}\right)}\right). \end{array}$$ (2)

Note that when *y*_*ij*_ = 1, *c*(1 − *y*_*ij*_) = 1 − *y*_*ij*_, and when *y*_*ij*_ = 0, *cy*_*ij*_ = *y*_*ij*_. Hence, we can rewrite Eq (2) as follows:

$$\begin{array}{ccc} p(Y|U,V)= & & \left(\prod_{1\leq i\leq m,1\leq j\leq n,y_{ij}=1}p_{ij}^{cy_{ij}}{\left(1-p_{ij}\right)}^{\left(1-y_{ij}\right)}\right)\times \\ & & \left(\prod_{1\leq i\leq m,1\leq j\leq n,y_{ij}=0}p_{ij}^{cy_{ij}}{\left(1-p_{ij}\right)}^{\left(1-y_{ij}\right)}\right)\\ = & & \prod_{i=1}^{m}\prod_{j=1}^{n}p_{ij}^{cy_{ij}}{\left(1-p_{ij}\right)}^{\left(1-y_{ij}\right)}. \end{array}$$ (3)

In addition, we also place zero-mean spherical Gaussian priors on the latent vectors of drugs and targets as:

$$\begin{array}{c} p\left(U|\sigma_{d}^{2}\right)=\prod_{i=1}^{m}N\left(u_{i}|0,\sigma_{d}^{2}I\right),\;\;p\left(V|\sigma_{t}^{2}\right)=\prod_{j=1}^{n}N\left(v_{j}|0,\sigma_{t}^{2}I\right), \end{array}$$ (4)

where $\sigma_{d}^{2}$ and $\sigma_{t}^{2}$ are parameters controlling the variances of Gaussian distributions, and **I** denotes the identity matrix. Hence, through a Bayesian inference, we have

$$\begin{array}{c} p\left(U,V|Y,\sigma_{d}^{2},\sigma_{t}^{2}\right)\propto p\left(Y|U,V\right)p\left(U|\sigma_{d}^{2}\right)p\left(V|\sigma_{t}^{2}\right). \end{array}$$ (5)

The log of the posterior distribution is thus derived as follows:

$$\begin{array}{ccc} \log p(U,V|Y,\sigma_{d}^{2},\sigma_{t}^{2}) & = & \sum_{i=1}^{m}\sum_{j=1}^{n}cy_{ij}u_{i}v_{j}^{⊤}-\left(1+cy_{ij}-y_{ij}\right)\log\left[1+\exp(u_{i}v_{j}^{⊤})\right]\\ & & -\frac{1}{2\sigma_{d}^{2}}\sum_{i=1}^{m}∥u_{i}{∥}_{2}^{2}-\frac{1}{2\sigma_{t}^{2}}\sum_{j=1}^{n}{∥v_{j}∥}_{2}^{2}+C, \end{array}$$ (6)

where *C* is a constant term independent of the model parameters (i.e., **U** and **V**). The model parameters can then be learned by maximizing the posterior distribution, which is equivalent with minimizing the following objective function:

$$\begin{array}{c} \min_{U,V}\sum_{i=1}^{m}\sum_{j=1}^{n}\left(1+cy_{ij}-y_{ij}\right)\log\left[1+\exp(u_{i}v_{j}^{⊤})\right]-cy_{ij}u_{i}v_{j}^{⊤}+\frac{\lambda_{d}}{2}{∥U∥}_{F}^{2}+\frac{\lambda_{t}}{2}{∥V∥}_{F}^{2}, \end{array}$$ (7)

where $\lambda_{d}=\frac{1}{\sigma_{d}^{2}}$, $\lambda_{t}=\frac{1}{\sigma_{t}^{2}}$, and ‖⋅‖_*F*_ denotes the Frobenius norm of a matrix. The problem in Eq (7) can be solved using an alternating gradient descent method [35].

### Regularized by Neighborhood

Through mapping both drugs and targets into a shared latent space, the LMF model can effectively estimate the global structure of the DTI data. However, LMF ignores the strong neighborhood associations among a small set of closely related drugs or targets. Thus, we propose to exploit the nearest neighborhood of a drug and that of a target to further improve the DTI prediction accuracy. For a drug *d*_*i*_, we denote the set of its nearest neighbors by *N*(*d*_*i*_) ∈ *D*\*d*_*i*_, where *N*(*d*_*i*_) is constructed by choosing *K*_1_ most similar drugs with *d*_*i*_. Then, we construct the set *N*(*t*_*j*_) ∈ *T*\*t*_*j*_, which consists of the *K*_1_ most similar targets with *t*_*j*_. In the experiments, we empirically set *K*_1_ to 5.

In this paper, the drug neighborhood information is represented using an adjacency matrix **A**, where the (*i*, *μ*) element *a*_*iμ*_ is defined as follows:

$$\begin{array}{c} a_{i\mu }=\left\{\begin{array}{cc} s_{i\mu }^{d} & \;\;\;\text{if}\;d_{\mu }\in N\left(d_{i}\right)\\ 0 & \;\;\;\text{otherwise.} \end{array}\right. \end{array}$$ (8)

Similarly, the adjacency matrix used to describe the target neighborhood information is denoted by **B**, where its (*j*, *ν*) element *b*_*jν*_ is defined as follows:

$$\begin{array}{c} b_{j\nu }=\left\{\begin{array}{cc} s_{j\nu }^{t} & \;\;\;\text{if}\;t_{\nu }\in N\left(t_{j}\right)\\ 0 & \;\;\;\text{otherwise.} \end{array}\right. \end{array}$$ (9)

Note that the adjacency matrices **A** and **B** are not symmetric.

The primary idea of exploiting the drug neighborhood information for DTI prediction is to minimize the distances between *d*_*i*_ and its nearest neighbors *N*(*d*_*i*_) in the latent space. This objective can be achieved by minimizing the following objective function:

$$\begin{array}{ccc} & & \frac{\alpha }{2}\sum_{i=1}^{m}\sum_{\mu =1}^{m}a_{i\mu }{∥u_{i}-u_{\mu }∥}_{F}^{2}\\ = & & \frac{\alpha }{2}\left[\sum_{i=1}^{m}\left(\sum_{\mu =1}^{m}a_{i\mu }\right)u_{i}u_{i}^{⊤}+\sum_{\mu =1}^{m}\left(\sum_{i=1}^{m}a_{i\mu }\right)u_{\mu }u_{\mu }^{⊤}\right]-\frac{\alpha }{2}\text{tr}\left(U^{⊤}AU\right)-\frac{\alpha }{2}\text{tr}\left(U^{⊤}A^{⊤}U\right)\\ = & & \frac{\alpha }{2}\text{tr}\left(U^{⊤}L^{d}U\right), \end{array}$$ (10)

where *tr*(⋅) is the trace of a matrix, $L^{d}=\left(D^{d}+{\tilde{D}}^{d}\right)-\left(A+A^{⊤}\right)$. **D**^*d*^ and ${\tilde{D}}^{d}$ are two diagonal matrices, in which the diagonal elements are $D_{ii}^{d}=\sum_{\mu =1}^{m}a_{i\mu }$ and ${\tilde{D}}_{\mu \mu }^{d}=\sum_{i=1}^{m}a_{i\mu }$ respectively. Moreover, we also exploit the neighborhood information of targets for DTI prediction by minimizing the following objective function:

$$\begin{array}{c} \frac{\beta }{2}\sum_{j=1}^{n}\sum_{\nu =1}^{n}b_{j\nu }{∥v_{j}-v_{\nu }∥}_{F}^{2}=\frac{\beta }{2}\text{tr}\left(V^{⊤}L^{t}V\right), \end{array}$$ (11)

where $L^{t}=\left(D^{t}+{\tilde{D}}^{t}\right)-\left(B+B^{⊤}\right)$, **D**^*t*^ and ${\tilde{D}}^{t}$ are two diagonal matrices, in which the diagonal elements are $D_{jj}^{t}=\sum_{\nu =1}^{n}b_{j\nu }$ and ${\tilde{D}}_{\nu \nu }^{d}=\sum_{j=1}^{n}b_{j\nu }$. Note that the proposed neighborhood regularization only considers influences from the *K*_1_ nearest neighbors of each drug and each target. It is different from the graph Laplacian constraints used in previous studies [38, 39] which consider influences from all similar drugs and targets. Clearly, given a drug-target pair, we leverage their nearest neighbors, instead of all the neighbors that could potentially introduce noisy information, to enhance the prediction accuracy.

### NRLMF

The final DTI prediction model can be formulated by considering the drug-target interactions as well as the neighborhood of drugs and targets. By plugging Eqs (10) and (11) into Eq (7), the proposed NRLMF model is formulated as follows:

$$\begin{array}{c} \min_{U,V}\sum_{i=1}^{m}\sum_{j=1}^{n}\left(1+cy_{ij}-y_{ij}\right)\ln\left[1+\exp(u_{i}v_{j}^{⊤})\right]-cy_{ij}u_{i}v_{j}^{⊤}\\ \;\;\;\;+\frac{1}{2}\text{tr}\left[U^{⊤}\left(\lambda_{d}I+\alpha L^{d}\right)U\right]+\frac{1}{2}\text{tr}\left[V^{⊤}\left(\lambda_{t}I+\beta L^{t}\right)V\right]. \end{array}$$ (12)

The optimization problem in Eq (12) can be solved by an alternating gradient ascent procedure. Denoting the objective function in Eq (12) by *L*, the partial gradients with respect to **U** and **V** are as follows:

$$\begin{array}{ccc} \frac{\partial L}{\partial U} & = & PV+\left(c-1\right)\left(Y⊙P\right)V-cYV+\left(\lambda_{d}I+\alpha L^{d}\right)U\\ \frac{\partial L}{\partial V} & = & P^{⊤}U+\left(c-1\right)\left(Y^{⊤}⊙P^{⊤}\right)U-cY^{⊤}U+\left(\lambda_{t}I+\beta L^{t}\right)V, \end{array}$$ (13)

where $P\in R^{m\times n}$, in which the (*i*, *j*) element is *p*_*ij*_ (see Eq (1)), ⊙ denotes the Hadamard product of two matrices. To accelerate the convergence of the gradient descent optimization methods, we use the AdaGrad algorithm [40] to adaptively choose the gradient step size. The details of the optimization algorithm to the proposed NRLMF model are described in Algorithm 1, where **U** and **V** are randomly initialized using a Gaussian distribution with mean 0, standard deviation $\frac{1}{\sqrt{r}}$.

**Algorithm 1: NRLMF**

 **Input**: **Y**, **S**^*d*^, **S**^*t*^, *c*, *r*, *K*_1_, *K*_2_, *λ*_*d*_, *λ*_*t*_, *α*, *β*, *γ*

 **Output**: **U**, **V**

1 Initialize **U** and **V** randomly, and set *φ*_*ik*_ = 0, *ϕ*_*jk*_ = 0, ∀1 ≤ *i* ≤ *m*, 1 ≤ *j* ≤ *n*, and 1 ≤ *k* ≤ *r*;

2 Construct the adjacency matrices **A** and **B** according to Eq (8) and Eq (9) respectively;

3 Compute the neighborhood regularization matrices **L**^*d*^ and **L**^*t*^ according to Eq (10) and Eq (11) respectively;

4 **for** *t* = 1, …, *max*_*iter* **do**

5  $G^{d}\leftarrow \frac{\partial L}{\partial U}$; // fix **V** and compute the gradient with respect to **U**

6  **for** *i* = 1, …, *m* **do**

7   **for** *k* = 1, …, *r* **do**

   // $g_{ik}^{d}$ and *u*_*ik*_ are the (*i*, *k*) element in **G**^*d*^ and **U** respectively.

8    $\varphi_{ik}\leftarrow \varphi_{ik}+g_{ik}^{d}\cdot g_{ik}^{d}$

9    $u_{ik}\leftarrow u_{ik}-\gamma \frac{g_{ik}^{d}}{\sqrt{\varphi_{ik}}}$; // update each element of *d*_*i*_’s latent vector

10  $G^{t}\leftarrow \frac{\partial L}{\partial V}$; // fix **U** and compute the gradient with respect to **V**

11  **for** *j* = 1, …, *n* **do**

12   **for** *k* = 1, …, *r* **do**

   // $g_{jk}^{t}$ and *v*_*jk*_ are the (*j*, *k*) element in **G**^*t*^ and **V** respectively.

13    $\phi_{jk}\leftarrow \phi_{jk}+g_{jk}^{t}\cdot g_{jk}^{t}$

14    $v_{jk}\leftarrow v_{jk}-\gamma \frac{g_{jk}^{t}}{\sqrt{\phi_{jk}}}$; // update each element of *t*_*j*_’s latent vector

Once the latent vectors **U** and **V** have been learned, the probability associated with any unknown drug-target pair (*d*_*i*_, *t*_*j*_) can be predicted by Eq (1). However, in the training procedure, the latent vectors of drugs belonging to the negative drug set *D*^−^ and those of the targets belonging to the negative target set *T*^−^ are learned solely based on negative observations (i.e., unknown pairs). As we know, some negative observations may be potential positive DTIs. Due to such uncertainty over negative observations, the learned latent vectors of the negative drugs and targets may not be accurate enough to describe their properties. One remedy for this problem is to replace the latent vector of a negative drug/target using the linear combination of the latent vectors of its nearest neighbors in the positive set. For a drug *d*_*i*_ ∈ *D*^−^, we denote the set of its *K*_2_ nearest neighbors in *D*^+^ by *N*^+^(*d*_*i*_). Similarly, for a target *t*_*j*_ ∈ *T*^−^, the set of its *K*_2_ nearest neighbors in *T*^+^ is denoted by *N*^+^(*t*_*j*_). Note that *N*^+^(*d*_*i*_) and *N*^+^(*t*_*j*_) are built using the same criteria as that used to construct the neighborhood in the training procedure. Then, the prediction of the interaction probability of a drug-target pair (*u*_*i*_, *v*_*j*_) is modified as,

$$\begin{array}{c} {\hat{p}}_{ij}=\frac{\exp({\tilde{u}}_{i}{\tilde{v}}_{j}^{⊤})}{1+\exp({\tilde{u}}_{i}{\tilde{v}}_{j}^{⊤})}, \end{array}$$ (14)

where

$$\begin{array}{cc} & {\tilde{u}}_{i}=\left\{\begin{array}{cc} u_{i}\;\;\; & \text{if}\;d_{i}\in D^{+}\\ \frac{1}{\sum_{\mu \in N^{+}\left(d_{i}\right)}s_{i\mu }^{d}}\sum_{\mu \in N^{+}\left(d_{i}\right)}s_{i\mu }^{d}u_{\mu }\;\;\; & \text{if}\;d_{i}\in D^{-}\text{,} \end{array}\right.\\ & {\tilde{v}}_{j}=\left\{\begin{array}{cc} v_{j}\;\;\; & \text{if}\;t_{j}\in T^{+}\\ \frac{1}{\sum_{\nu \in N^{+}\left(t_{j}\right)}s_{j\nu }^{t}}\sum_{\nu \in N^{+}\left(t_{j}\right)}s_{j\nu }^{t}v_{\nu }\;\;\; & \text{if}\;t_{j}\in T^{-}\text{.} \end{array}\right. \end{array}$$ (15)

Note that Eq (15) shows a general case for smoothing the learned drug-specific and target-specific latent vectors. In the experiments, *K*_2_ is empirically set to 5 to simplify the model.

## Results

We have performed extensive experiments to evaluate the performance of the proposed NRLMF method.

### Experimental Settings

Following previous studies [13–15, 19, 24, 27], the performance of the DTI prediction methods were evaluated under five trials of 10-fold cross-validation (CV), and both AUC and AUPR were used as the evaluation metrics. In particular, for each method, we performed 10-fold CV for five times, each time with a different random seed. Then, we calculated an AUC score in each repetition of CV and reported a final AUC score that was the average over the five repetitions. The AUPR score was calculated in the same manner.

The drug-target interaction matrix $Y\in R^{m\times n}$ had *m* rows for drugs and *n* columns for targets. We conducted CV under three different settings as follows [19, 27, 41].

- CVS1: CV on drug-target pairs—random entries in **Y** (i.e., drug-target pairs) were selected for testing.
- CVS2: CV on drugs—random rows in **Y** (i.e., drugs) were blinded for testing.
- CVS3: CV on targets—random columns in **Y** (i.e., targets) were blinded for testing.

Under CVS1, in each round, we used 90% of elements in **Y** as training data and the remaining 10% of elements as test data. Under CVS2, in each round, we used 90% of rows in **Y** as training data and the remaining 10% of rows as test data. Under CVS3, in each round, we used 90% of columns in **Y** as training data and the remaining 10% of columns as test data. Note that these three settings CVS1, CVS2, and CVS3 refer to the DTI prediction for 1) new (unknown) pairs, 2) new drugs, and 3) new targets, respectively.

In this paper, we compared the proposed NRLMF method with the following state-of-the-art methods, namely, NetLapRLS [13], KBMF2K [24], BLM-NII [17], WNN-GIP [15], and CMF [27], by testing their prediction capabilities under the above three settings. The settings of the hyper-parameters of each method were as follows. For the matrix factorization based methods, the dimensionality of the latent space *r* was selected from {50, 100} [27]. In NRLMF, we set *λ*_*d*_ = *λ*_*t*_ and chose these two parameters from {2^−5^, 2^−4^, ⋯, 2^1^}. The neighborhood regularization parameters *α* and *β* of NRLMF were selected from {2^−5^, 2^−4^, ⋯, 2^2^} and {2^−5^, 2^−4^, ⋯, 2^0^}, respectively, and the optimal learning rate *γ* was selected from {2^−3^, 2^−2^, ⋯, 2^0^}. In KBMF2K, the margin parameter *ν* was selected from {0, 1}. For CMF, the regularization coefficient *λ*_*l*_ was chosen from {2^−2^, ⋯, 2^1^}, while *λ*_*d*_ and *λ*_*t*_ were chosen from {2^−3^, 2^−2^, ⋯, 2^5^}. For NetLapRLS, we set *γ*_*d*_2__/*γ*_*d*_1__ = *γ*_*p*_2__/*γ*_*p*_1__, *β*_*d*_ = *β*_*p*_, and chose their values from {10^−6^, 10^−5^, ⋯, 10^2^}. In BLM-NII, the linear combination weight *α* was chosen from {0.0, 0.1, ⋯, 1.0}, and the *max* function was used to integrate the interaction scores predicted independently from the drug side and the target side. For WNN-GIP, the decay value *T* was chosen from {0.1, 0.2, ⋯, 0.9}. We set the weighting parameters *α*_*d*_ = *α*_*t*_ and chose their values from {0.0, 0.1, ⋯, 1.0}. For a machine learning methods, the most suitable hyper-parameters on different datasets are usually different. Thus, we need to choose the optimal hyper-parameters for each method on different datasets. In the literature, the most widely used hyper-parameter optimization strategies are grid search and manual search [42]. In this work, we adopted grid search to choose the optimal hyper-parameters for each DTI prediction method on each dataset. As part of future work, we would like to use the random search strategy proposed in [42] to improve the efficiency of hyper-parameter optimization for DTI prediction methods.

### Comparisons with the State-of-the-Arts

Table 2 shows the AUC and AUPR values obtained by various methods under the setting CVS1. As shown in Table 2, NRLMF attains the best AUC values over all datasets. The final average AUC obtained by NRLMF is 0.974, which is 2.10% better than the second method BLM-NII. Moreover, NRLMF achieves the highest AUPR over three datasets (i.e., Nuclear Receptor, GPCR, and Enzyme) and obtains the second best AUPR on the Ion Channel dataset, where CMF outperforms NRLMF (0.923 for CMF vs. 0.906 for NRLMF). The average AUPR obtained by NRLMF is 0.819, which is 4.73% higher than that obtained by the second best method CMF. In summary, under the setting CVS1, NRLMF outperforms other competing methods, being statistically significant except two comparison cases with CMF at the significant level of 0.05 using t-test.

**Table 2:** The AUC and AUPR obtained under the setting CVS1.

AUC						
Dataset	NetLapRLS	BLM-NII	WNN-GIP	KBMF2K	CMF	NRLMF
Nuclear Receptor	0.850±0.021*	0.905±0.023*	0.901±0.017*	0.877±0.023*	0.864±0.026*	**0.950** ±0.011
GPCR	0.915±0.006*	0.950±0.006*	0.944±0.005*	0.926±0.006*	0.940±0.007*	**0.969** ±0.004
Ion Channel	0.969±0.003*	0.981±0.002*	0.959±0.003*	0.961±0.003*	0.981±0.002*	**0.989** ±0.001
Enzyme	0.972±0.002*	0.978±0.002*	0.964±0.003*	0.905±0.003*	0.969±0.002*	**0.987** ±0.001
Avg.	0.927	0.954	0.942	0.917	0.939	**0.974**
AUPR						
Nuclear Receptor	0.465±0.044*	0.659±0.039*	0.589±0.034*	0.534±0.050*	0.584±0.042*	**0.728** ±0.041
GPCR	0.616±0.015*	0.524±0.024*	0.520±0.021*	0.578±0.018*	0.745±0.013	**0.749** ±0.015
Ion Channel	0.837±0.009*	0.821±0.012*	0.717±0.020*	0.771±0.009*	**0.923** ±0.006	0.906±0.008
Enzyme	0.789±0.005*	0.752±0.011*	0.706±0.017*	0.654±0.008*	0.877±0.005*	**0.892** ±0.006
Avg.	0.677	0.689	0.633	0.634	0.782	**0.819**

“Avg.” shows the average AUC/AUPR over four datasets. The best results in each row are in **bold faces** and the second best results are underlined.

* indicates NRLMF significantly outperforms the competitor with *p* < 0.05 using t-test.

The results obtained under the setting CVS2 for new drugs are shown in Table 3. In particular, NRLMF outperforms the other methods over the Nuclear Receptor, GPCR, and Ion Channel datasets, in terms of AUC. On the Enzyme dataset, WNN-GIP achieves a little better AUC than NRLMF (0.882 for WNN-GIP vs. 0.871 for NRLMF). Over all datasets, NRLMF obtains the best average AUC value 0.870. For the AUPR metric, NRLMF achieves the best results on all datasets except the GPCR dataset, where KBMF2K and CMF are slightly better than NRLMF. Overall, NRLMF achieves the best average AUPR 0.403, which is 13.84% higher than the second-best method KBMF2K and 17.84% higher than the third-best method CMF.

**Table 3:** The AUC and AUPR obtained under the setting CVS2.

AUC						
Dataset	NetLapRLS	BLM-NII	WNN-GIP	KBMF2K	CMF	NRLMF
Nuclear Receptor	0.789±0.039*	0.799±0.037*	0.890±0.023	0.844±0.023*	0.818±0.036*	**0.900**±0.021
GPCR	0.817±0.015*	0.838±0.016*	0.891±0.010	0.839±0.020*	0.857±0.014*	**0.895**±0.011
Ion Channel	0.757±0.025*	0.796±0.025	0.797±0.028	0.799±0.019	0.743±0.029*	**0.813** ±0.027
Enzyme	0.786±0.023*	0.813±0.022*	**0.882**±0.015	0.713±0.029*	0.829±0.019*	0.871±0.017
Avg.	0.787	0.812	0.865	0.799	0.812	**0.870**
AUPR						
Nuclear Receptor	0.417±0.048*	0.438±0.048*	0.504±0.056	0.477±0.049	0.488±0.050	**0.545**±0.054
GPCR	0.229±0.017*	0.315±0.022*	0.295±0.025*	**0.366**±0.024	0.365±0.022	0.364±0.023
Ion Channel	0.200±0.026*	0.302±0.033	0.258±0.032*	0.308±0.038	0.286±0.030*	**0.344**±0.033
Enzyme	0.123±0.009*	0.253±0.023*	0.278±0.037*	0.263±0.033*	0.229±0.020*	**0.358**±0.040
Avg.	0.242	0.327	0.334	0.354	0.342	**0.403**

“Avg.” shows the average AUC/AUPR over four datasets. The best results in each row are in **bold faces** and the second best results are underlined.

* indicates NRLMF significantly outperforms the competitor with *p* < 0.05 using t-test.

In addition, Table 4 summarizes the results obtained under the setting CVS3 for new targets. We observe that WNN-GIP outperforms other methods on the Nuclear Receptor dataset, in terms of AUC and AUPR. On the other three datastes, the proposed NRLMF achieves the best AUC and AUPR values. Over all datasets, WNN-GIP achieves the highest average AUC value 0.940, which is 1.29% better than the second-best method NRLMF. For the AUPR measure, NRLMF achieves the best average AUPR 0.651, which is a 11.09% better than the second-best method WNN-GIP.

**Table 4:** The AUC and AUPR obtained under the setting CVS3.

AUC						
Dataset	NetLapRLS	BLM-NII	WNN-GIP	KBMF2K	CMF	NRLMF
Nuclear receptor	0.655±0.046*	0.534±0.086*	**0.935**±0.017	0.668±0.060*	0.680±0.066*	0.851±0.027
GPCR	0.770±0.024*	0.778±0.025*	0.926±0.013	0.882±0.016*	0.837±0.019*	**0.930**±0.012
Ion Channel	0.914±0.012*	0.914±0.012*	0.950±0.007*	0.938±0.008*	0.905±0.012*	**0.964**±0.007
Enzyme	0.905±0.014*	0.909±0.014*	0.947±0.008*	0.876±0.012*	0.915±0.013*	**0.966**±0.005
Avg.	0.811	0.784	**0.940**	0.841	0.834	0.928
AUPR						
Nuclear Receptor	0.449±0.074	0.402±0.083	**0.531**±0.073	0.324±0.071	0.400±0.077	0.449±0.079
GPCR	0.334±0.025*	0.341±0.034*	0.550±0.047	0.516±0.045	0.433±0.028*	**0.556**±0.038
Ion Channel	0.737±0.020*	0.762±0.020	0.696±0.035*	0.677±0.021*	0.620±0.027*	**0.785**±0.028
Enzyme	0.669±0.021*	0.735±0.022*	0.566±0.038*	0.565±0.023*	0.698±0.021*	**0.812**±0.018
Avg.	0.547	0.560	0.586	0.521	0.538	**0.651**

“Avg.” shows the average AUC/AUPR over four datasets. The best results in each row are in **bold faces** and the second best results are underlined.

* indicates NRLMF significantly outperforms the competitor with *p* < 0.05 using t-test.

The task under the setting CVS1 focuses on predicting the unknown pair (*d*_*i*_, *t*_*j*_), where at least one DTI is known for *d*_*i*_ and *t*_*j*_ respectively in the training data. However, the tasks under CVS2 and CVS3 focus on the predictions for new drugs and new targets respectively, where no DTIs are observed for new drugs and new targets in the training data. Therefore, the task under CVS1 is easier than those under CVS2 and CVS3, and the AUC and AUPR values obtained by DTI prediction methods under CVS1 are higher than those obtained under CVS2 and CVS3 as expected. For all CV settings, the proposed NRLMF method achieves the best AUC values in 10 out of 12 scenarios (i.e., 3 CV settings on 4 datasets) via integrating LMF with neighborhood regularization. In the remaining 2 scenarios (i.e., CVS2 on Enzyme dataset and CVS3 on Nuclear Receptor dataset), WNN-GIP attains better AUC values than NRLMF. The results in these 2 scenarios can be interpreted as follows. For instance, under CVS2, the interactions for 10% of the drugs (i.e., the set of negative drugs *D*^−^) have been blinded in the training phase. The latent vectors of *D*^−^ are learned solely based on negative observations, and thus are not accurate. This may lead to the inaccuracies of the learned latent vectors of targets (see Eq (13)). Especially, for the targets with only one interaction, the accuracies of the learned latent vectors may be drastically reduced. In NRLMF, the latent vectors of negative drugs and targets are smoothed using their nearest neighbors. However, there is no smoothing for the latent vectors of targets with only one interaction (see Eq (15)). As such, the performances of NRLMF under CVS2 may be affected more on the dataset with a higher percentage of targets that have only one interaction. Interestingly, over 4 datasets, the percentage of targets that have only one interaction is 30.77%, 35.79%, 11.27%, and 43.37%, for Nuclear Receptor, GPCR, Ion Channel, and Enzyme, respectively. Enzyme dataset has the highest percentage of targets with only one interaction, and thus the performance of NRLMF on this dataset under CVS2 is likely to be affected most. Similarly, the percentage of drugs with only one interaction is 72.22% for Nuclear Receptor, 47.53% for GPCR, 38.57% for Ion Channel, and 39.78% for Enzyme. Thus, by blinding the interactions of 10% targets (i.e., under CVS3), the performance of NRLMF on Nuclear Receptor dataset is the most likely to be affected. For the AUPR metric, NRLMF attains the best AUPR values in 9 out of 12 scenarios, which is to be expected, since the methods that optimize AUC are not guaranteed to optimize AUPR [43]. In addition, the target sequence similarity **S**^*t*^ is more reliable and informative than the drug chemical similarity **S**^*d*^[14]. Hence, the information propagated from the neighbors to the new targets by the regularization term in Eq (11) will be more accurate than those to new drugs by the term in Eq (10). This explains the results well that various methods usually achieve higher AUC and AUPR under CVS3 than CVS2.

### Neighborhood Benefits

The proposed NRLMF method incorporates neighborhood information for DTI prediction via the neighborhood regularization in training and the neighborhood smoothing in prediction. Next, we will study how the neighborhood information benefits DTI prediction under the setting CVS1. For the results under CVS2 and CVS3, please refer to the supporting S1–S8 Figs for details.

**Fig 1:**
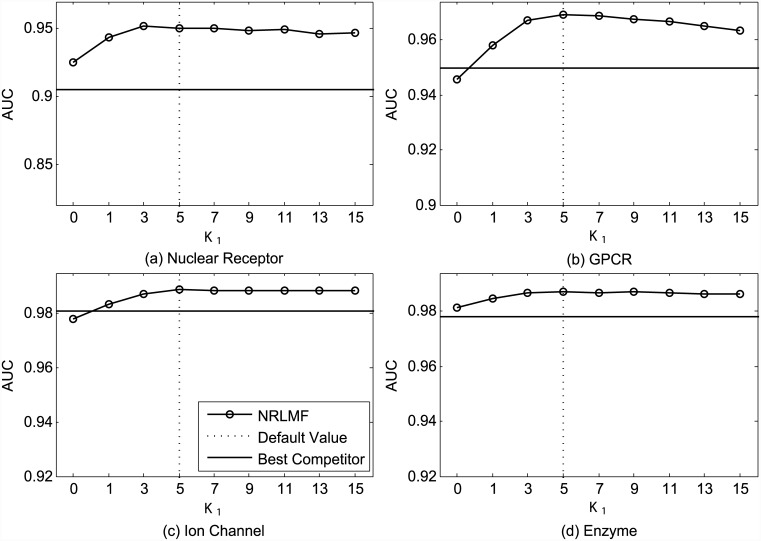
Performance trend of NRLMF on the benchmark datasets (a) Nuclear Receptor, (b) GPCR, (c) Ion Channel, and (d) Enzyme, measured by AUC with different settings of *K*_1_ under CVS1. The best competitors on these datasets are (a) BLM-NII, (b) BLM-NII, (c) BLM-NII and CMF, and (d) BLM-NII, respectively.

**Fig 2:**
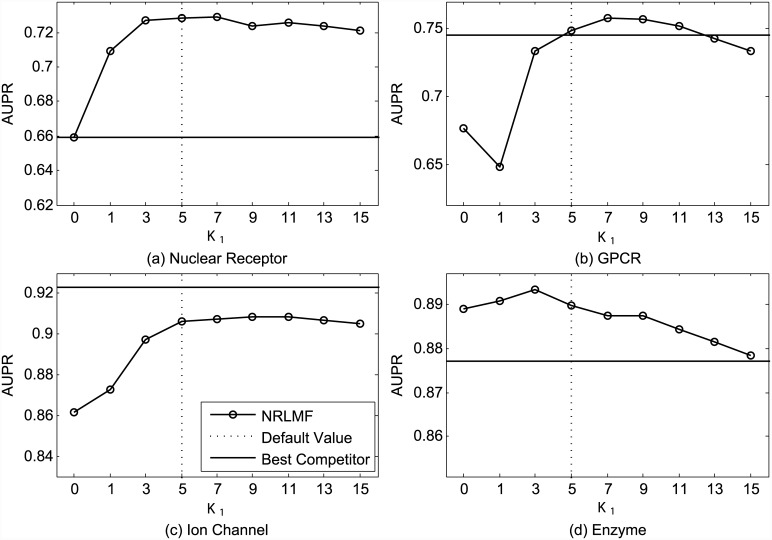
Performance trend of NRLMF on the benchmark datasets (a) Nuclear Receptor, (b) GPCR, (c) Ion Channel, and (d) Enzyme, measured by AUPR with different settings of *K*_1_ under CVS1. The best competitors on these datasets are (a) BLM-NII, (b) CMF, (c) CMF, and (d) CMF, respectively.

Fig 1 shows the AUC values obtained by NRLMF with respect to different settings of the neighborhood size *K*_1_ used for the neighborhood regularization in the training procedure. As shown in Fig 1, the optimal values of *K*_1_ are 3, 5, 5, and 5, for four datasets, respectively. Under the setting CVS1, the average AUC of NRLMF is 0.958 when *K*_1_ is set as 0 (i.e., without neighborhood regularization in training), while it is increased to 0.974 when *K*_1_ is set as 5. Fig 2 illustrates the AUPR values with respect to different settings of *K*_1_. We find that NRLMF achieves the best AUPR by setting *K*_1_ as 7, 7, 9, and 3, respectively. When *K*_1_ = 0, the average AUPR achieved by NRLMF without neighborhood regularization is 0.772, while it is increased to 0.818 by setting *K*_1_ = 5. These results highlight that the neighborhood regularization is highly desirable for DTI prediction.

**Fig 3:**
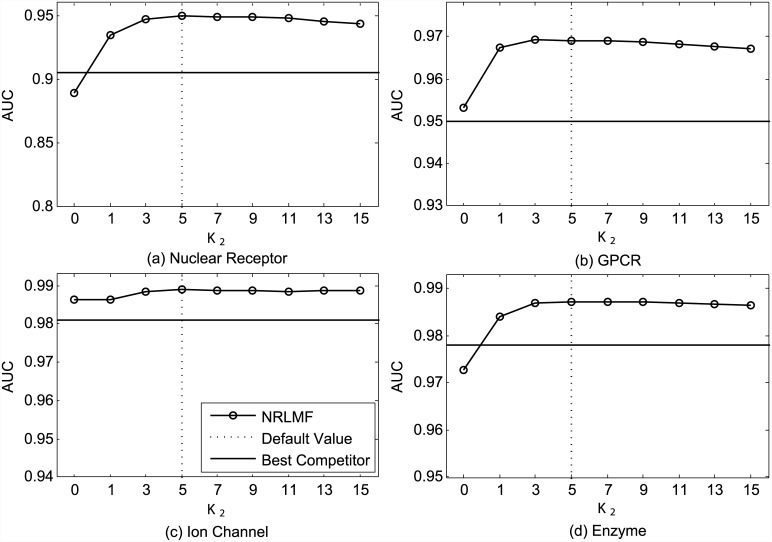
Performance trend of NRLMF on the benchmark datasets (a) Nuclear Receptor, (b) GPCR, (c) Ion Channel, and (d) Enzyme, measured by AUC with different settings of *K*_2_ under CVS1. The best competitors on these datasets are (a) BLM-NII, (b) BLM-NII, (c) BLM-NII and CMF, and (d) BLM-NII, respectively.

**Fig 4:**
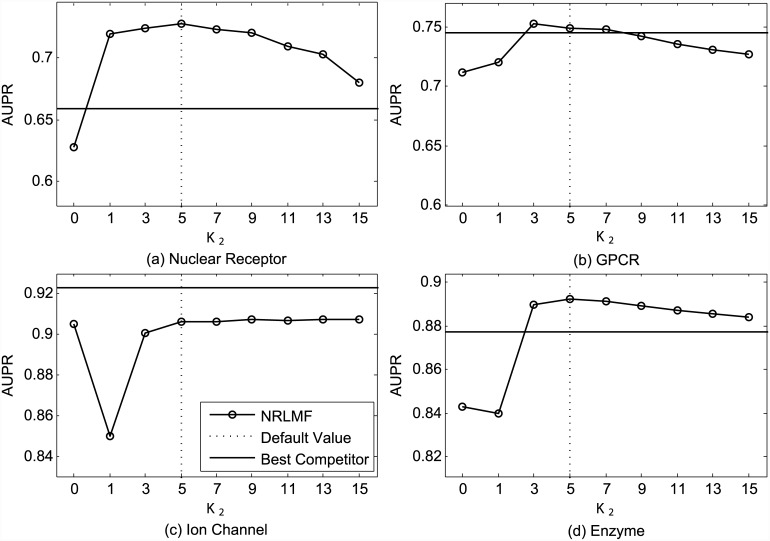
Performance trend of NRLMF on the benchmark datasets (a) Nuclear Receptor, (b) GPCR, (c) Ion Channel, and (d) Enzyme, measured by AUPR with different settings of *K*_2_ under CVS1. The best competitors on these datasets are (a) BLM-NII, (b) CMF, (c) CMF, and (d) CMF, respectively.

In addition, we also study the impact of the neighborhood size *K*_2_ used for neighborhood smoothing in the prediction procedure. Figs 3 and 4 plot the AUC and AUPR values obtained by NRLMF with respect to different settings of *K*_2_. As shown in Fig 3, NRLMF achieves best AUC via setting *K*_2_ as 5, 3, 5, and 5, respectively. For AUPR measure, the best results are achieved by setting *K*_2_ as 5, 3, 9, and 5, respectively. Over all datasets, when *K*_2_ = 0 (i.e., without neighborhood smoothing in prediction), the average AUC and AUPR values obtained by NRLMF are 0.950 and 0.772, respectively, while these values are 0.974 and 0.819 when *K*_2_ = 5. These observations demonstrate the effectiveness of nearest neighbors to predict the interaction probability for a given drug-target pair. In addition, when we set *K*_1_ and *K*_2_ as 5, we can get reasonably good results for both AUC and AUPR, respectively.

### Parameter Sensitivity Analysis for *c* and *r*

In this section, we focus on the sensitivity analysis for other two parameters, i.e., the importance levels of observed DTIs *c* and the dimensionality of the latent space *r*, under the setting CVS1. As to the performance trend of NRLMF with respect to different settings for *c* and *r* under CVS2 and CVS3, please refer to the supporting S9–S16 Figs for details.

**Fig 5:**
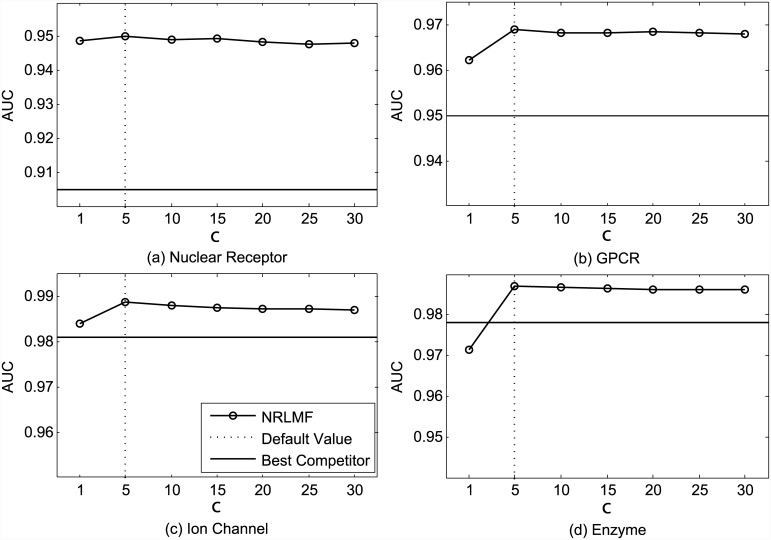
Performance trend of NRLMF on the benchmark datasets (a) Nuclear Receptor, (b) GPCR, (c) Ion Channel, and (d) Enzyme, measured by AUC with different settings of *c* under CVS1. The best competitors on these datasets are (a) BLM-NII, (b) BLM-NII, (c) BLM-NII and CMF, and (d) BLM-NII, respectively.

**Fig 6:**
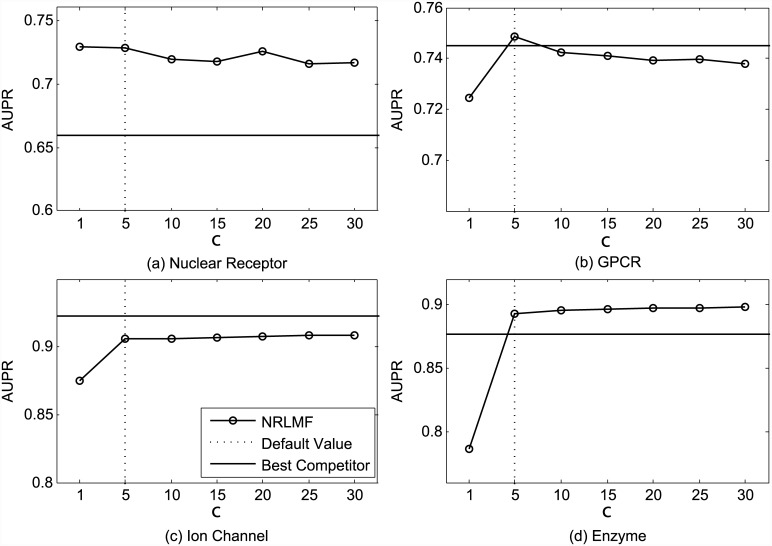
Performance trend of NRLMF on the benchmark datasets (a) Nuclear Receptor, (b) GPCR, (c) Ion Channel, and (d) Enzyme, measured by AUPR with different settings of *c* under CVS1. The best competitors on these datasets are (a) BLM-NII, (b) CMF, (c) CMF, and (d) CMF, respectively.

As shown in Fig 5, when the importance level *c* is set as 1 (i.e., without importance weighting), NRLMF outperforms other competitors on Nuclear Receptor, GPCR, and Ion Channel datasets, and is comparable with the best competitor on the Enzyme dataset (0.971 for NRLMF vs. 0.978 for the best competitor), in terms of AUC. This again highlights the effectiveness of integrating logistic matrix factorization with neighborhood regularization for DTI prediction. By setting *c* = 5, NRLMF is able to achieve the optimal AUC values and outperforms all competing methods over all datasets. For the AUPR metric, Fig 6 shows that NRLMF with setting *c* = 1 outperforms other competitors on the Nuclear Receptor dataset and performs poorer than the best competitor on the remaining three datasets. This is expected, since the methods that optimize AUC are not guaranteed to optimize AUPR [43]. In addition, NRLMF achieves better AUPR under the setting *c* > 1 than under the setting *c* = 1, on the GPCR, Ion Channel, and Enzyme datasets. On the Nuclear Receptor dataset, NRLMF attains slightly better AUPR under the setting *c* = 1 than under the other settings. These observations demonstrate that assigning more importance on the observed interactions can boost the performance of NRLMF. However, when *c* is large enough, the performance of NRLMF tends to become saturated, where further increasing *c* has very limited improvement.

**Fig 7:**
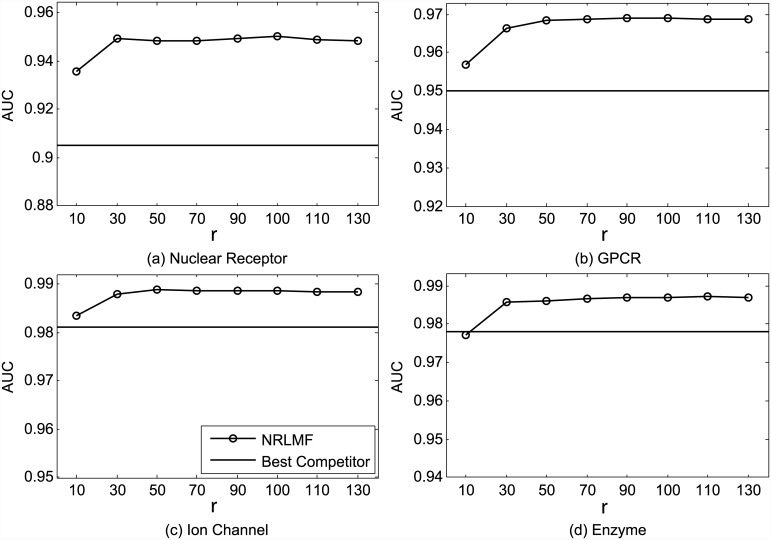
Performance trend of NRLMF on the benchmark datasets (a) Nuclear Receptor, (b) GPCR, (c) Ion Channel, and (d) Enzyme, measured by AUC with different settings of *r* under CVS1. The best competitors on these datasets are (a) BLM-NII, (b) BLM-NII, (c) BLM-NII and CMF, and (d) BLM-NII, respectively.

**Fig 8:**
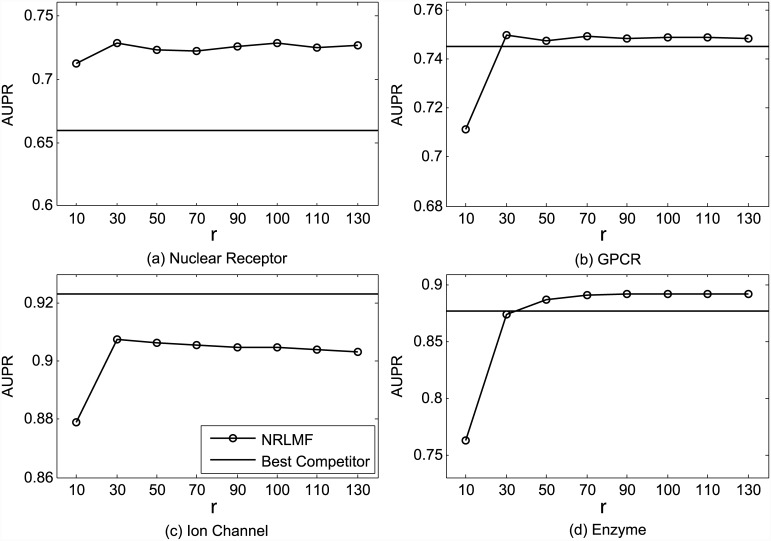
Performance trend of NRLMF on the benchmark datasets (a) Nuclear Receptor, (b) GPCR, (c) Ion Channel, and (d) Enzyme, measured by AUPR with different settings of *r* under CVS1. The best competitors on these datasets are (a) BLM-NII, (b) CMF, (c) CMF, and (d) CMF, respectively.

The impact of the dimensionality of the latent space *r* on the performance of NRLMF, in terms of AUC and AUPR, is shown in Figs 7 and 8, respectively. We find that larger *r* generally achieves better results. The two exceptions are the AUPR measure on Nuclear Receptor and Ion Channel datasets, where *r* = 30 leads to slightly better results than *r* = 50. Nevertheless, *r* = 100 achieves the best results or the second best results measured by AUC and AUPR, on all datasets. Thus, the parameter *r* is recommended to be set in the range [50, 100], which is consistent with previous studies [27].

### Predicting Novel Interactions

In this section, we evaluate the practical ability of NRLMF on predicting novel interactions, which refer to interactions with high probabilities that do not occur in the benchmark datasets. Following similar settings in previous studies [12, 14, 15, 19, 24], four well-known biological databases, i.e., ChEMBL [2], DrugBank [30], KEGG [4], and Matador [5], are used as references to verify whether the predicted new DTIs are true or not.

To conduct this study, we have collected the online profiles associated with the drugs and targets in each benchmark dataset from the online reference databases and parsed the approved drug-target interactions. Over all benchmark datasets, there are 791 drugs and 986 targets, and 1,999 novel interactions have been confirmed in one or more reference databases. The number of confirmed novel interactions in Nuclear Receptor, GPCR, Ion Channel, and Enzyme datasets are 21, 512, 1034, and 432, respectively. For each dataset, the entire dataset is used as training set. The unknown interactions will be ranked based on the interaction probabilities predicted using the optimal parameters learned under CVS1 instead of those learned under other two settings (i.e., CVS2 and CVS3). This is because that our objective is to predict those novel likely drug-target interactions, instead of focusing a new drug or a new target. Then, the predicted novel interactions are the top ranked unknown drug-target interaction pairs.

Table 5 shows the top 30 novel interactions predicted by NRLMF on the GPCR dataset. In this table, the DTIs are bolded to indicate that they exist in one or more of the reference databases. The third column of Table 5 shows the predicted interaction probability of a drug-target pair. For each pair, the databases containing it are listed in the last column of the table, where **C** is short for ChEMBL, **D** for DrugBank, **K** for KEGG, and **M** for Matador. For example, the highest ranked DTI is (D00283, hsa1814) with predicted probability 0.9181, which exists in the databases ChEMBL, DrugBank, and Metador. We find that 67% of the predictions (20 out of 30) are currently confirmed in at least one of the reference databases. Since these databases are still being updated as new DTIs are found, the fraction of new DTIs correctly predicted by NRLMF may increase in the future. This encouraging result that NRLMF can successfully detect quite a few novel interactions that are not in the GPCR dataset, implies that NRLMF is very effective in predicting new true DTIs from sparse matrices consisted of very few DTIs.

**Table 5:** The top 30 novel interactions predicted by NRLMF on GPCR dataset.

Rank	Drug	Target	Probability	Databases			
1	**D00283**	**hsa1814**	0.9181	**C**	**D**		**M**
2	**D02358**	**hsa154**	0.8828		**D**		
3	**D04625**	**hsa154**	0.8550		**D**	**K**	
4	D02614	hsa154	0.8373				
5	**D00227**	**hsa136**	0.8370	**C**			
6	**D01712**	**hsa136**	0.7736		**D**		
7	D01352	hsa5731	0.7718				
8	**D02250**	**hsa6751**	0.7611			**K**	
9	D02884	hsa136	0.7605				
10	D02354	hsa1814	0.7581				
11	**D02147**	**hsa153**	0.7500		**D**		**M**
12	D01871	hsa3269	0.7266				
13	**D00371**	**hsa134**	0.7064	**C**	**D**	**K**	
14	**D00371**	**hsa135**	0.7012	**C**	**D**	**K**	
15	D02725	hsa5737	0.6904				
16	D00682	hsa5739	0.6852				
17	**D04006**	**hsa135**	0.6803		**D**	**K**	
18	**D00049**	**hsa8843**	0.6659		**D**		
19	**D04006**	**hsa134**	0.6606		**D**	**K**	
20	**D00604**	**hsa147**	0.6603		**D**		
21	**D00715**	**hsa1129**	0.6584		**D**	**K**	
22	D00503	hsa3356	0.6538				
23	**D01103**	**hsa1129**	0.6417			**K**	
24	D02359	hsa153	0.6367				
25	**D00079**	**hsa5731**	0.6117	**C**	**D**		
26	D00765	hsa1128	0.6089				
27	**D02340**	**hsa1812**	0.6063		**D**		
28	**D00442**	**hsa6753**	0.5940			**K**	
29	**D00397**	**hsa1131**	0.5829	**C**	**D**	**K**	
30	**D00095**	**hsa155**	0.5797	**C**	**D**	**K**	

The confirmed drug-target interaction pairs are in **bold faces**.

Finally, Table 6 summarizes the fractions of true DTIs among the top *N* (*N* = 10, 30, 50) predictions generated by various DTI methods, using the optimal parameters learned under CVS1. We observe that NRLMF is able to achieve consistently accurate prediction results across all the datasets. For example, the fractions of true DTIs among the top 10 predicted interactions are 50%, 60%, 50%, and 90% for all datasets, respectively. Compared with other methods, NRLMF is able to achieve comparable or even better prediction results across all the datasets. These observations indicate that the proposed algorithm is very effective for finding novel DTIs, thus it may help biologists or clinicians significantly reduce the cost of biological test. For more details about the novel DTI prediction, please refer to the supporting S1–S4 Texts, where the top 1000 novel DTIs predicted by NRLMF are provided.

**Table 6:** The fractions of true DTIs among the predicted top *N* (*N* = 10, 30, 50) interactions under CVS1.

	Nuclear Receptor			GPCR			Ion Channel			Enzyme		
	Top 10	Top 30	Top 50	Top 10	Top 30	Top 50	Top 10	Top 30	Top 50	Top 10	Top 30	Top 50
NetLapRLS	10%	23%	26%	40%	40%	46%	60%	47%	38%	70%	50%	40%
BLM-NII	30%	27%	16%	70%	60%	58%	30%	30%	34%	70%	**60%**	**46%**
WNN-GIP	0%	20%	14%	30%	43%	38%	30%	43%	48%	70%	50%	40%
KBMF2K	40%	30%	22%	**90%**	53%	52%	**100%**	**83%**	**84%**	70%	43%	28%
CMF	10%	20%	24%	50%	40%	36%	0%	0%	6%	20%	7%	4%
NRLMF	**50%**	**43%**	**28%**	60%	**67%**	**60%**	50%	33%	34%	**90%**	**60**%	44%

## Discussion

This paper presents a novel drug-target interaction prediction method, namely neighborhood regularized logistic matrix factorization (NRLMF). The novelty of NRLMF comes from integrating logistic matrix factorization with neighborhood regularization to predict the interaction probability of a given drug-target pair. Specifically, both drugs and targets are mapped into a shared latent space, and the drug-target interactions are modeled using the linear combinations of the drug-specific and target-specific latent vectors. In addition, higher importance level is assigned to the positive observations (i.e., interaction pairs), while lower level is for negative observations (i.e., unknown pairs). Moreover, the neighborhood regularization based on the drug similarities and target similarities is utilized to further improve the prediction ability of the model.

To evaluate the performance of NRLMF, an extensive set of experiments were performed on four benchmark datasets, compared with five state-of-the-art DTI prediction methods. The promising results further validated the empirical efficacy of the proposed algorithm. For example, on average, NRLMF attains the best AUC values under CVS1 and CVS2, and the second best AUC value under CVS3. In terms of AUPR, NRLMF achieves the best averaged AUPR values over all datasets, under all three CV settings. These results indicate that NRLMF outperforms existing state-of-the-art methods in predicting new pairs and new drugs, and is comparable or even better than existing methods in predicting new targets. However, on the dataset with a large fraction of drugs which have only one interaction (e.g., 72.22% on the Nuclear Receptor dataset), WNN-GIP may outperform NRLMF in predicting new targets. On the dataset with a large fraction of targets which have only one interaction (e.g., 43.37% on the Enzyme dataset), WNN-GIP may achieve better results than NRLMF in predicting new drugs. In addition, the high practical predicting ability of NRLMF have also been verified. For example, on the Enzyme dataset, 90% of the top 10 novel DTIs predicted by NRLMF have been confirmed by the latest version of four well-known biological databases, including ChEMBL, DrugBank, KEGG, and Matador.

The optimization problem of NRLMF is solved using an alternating gradient descent optimization algorithm, the time complexity of which is *O*(*iter* ⋅ *r* ⋅ *m* ⋅ *n*), where *iter* denotes the number of iterations. However, the time complexity of the solutions to the other two matrix factorization based DTI prediction methods (i.e., KBMF2K and CMF) are *O*(*iter* ⋅ (*r* ⋅ *m*^3^+*r* ⋅ *n*^3^+*r*^3^)) and *O*(*iter* ⋅ (*r*^2^ ⋅ (*m*+*n*)^2^+*r*^3^ ⋅ (*m*+*n*))), respectively. Therefore, NRLMF is more efficient than KBMF2K and CMF. In addition, NRLMF can also be extended to incorporate multiple types of similarities from drugs and targets for DTI prediction. One direction for future work is to couple logistic matrix factorization with the multiple kernel learning techniques [44]. Another potential direction for future work is to exploit boosting technique, e.g., the AdaBPR model in [45], to improve the prediction accuracy of the proposed NRLMF method.

## Supporting Information

S1 Fig Performance trend of NRLMF on the benchmark datasets (a) Nuclear Receptor, (b) GPCR, (c) Ion Channel, and (d) Enzyme, measured by AUC with different settings of *K*_1_ under CVS2. The best competitors on these datasets are (a) WNN-GIP, (b) WNN-GIP, (c) KBMF2K, and (d) WNN-GIP, respectively.
(EPS)

S2 Fig Performance trend of NRLMF on the benchmark datasets (a) Nuclear Receptor, (b) GPCR, (c) Ion Channel, and (d) Enzyme, measured by AUPR with different settings of *K*_1_ under CVS2. The best competitors on these datasets are (a) WNN-GIP, (b) KBMF2K, (c) KBMF2K, and (d) WNN-GIP, respectively.
(EPS)

S3 Fig Performance trend of NRLMF on the benchmark datasets (a) Nuclear Receptor, (b) GPCR, (c) Ion Channel, and (d) Enzyme, measured by AUC with different settings of *K*_1_ under CVS3. The best competitors on these datasets are (a) WNN-GIP, (b) WNN-GIP, (c) WNN-GIP, and (d) WNN-GIP, respectively.
(EPS)

S4 Fig Performance trend of NRLMF on the benchmark datasets (a) Nuclear Receptor, (b) GPCR, (c) Ion Channel, and (d) Enzyme, measured by AUPR with different settings of *K*_1_ under CVS3. The best competitors on these datasets are (a) WNN-GIP, (b) WNN-GIP, (c) BLM-NII, and (d) BLM-NII, respectively.
(EPS)

S5 Fig Performance trend of NRLMF on the benchmark datasets (a) Nuclear Receptor, (b) GPCR, (c) Ion Channel, and (d) Enzyme, measured by AUC with different settings of *K*_2_ under CVS2. The best competitors on these datasets are (a) WNN-GIP, (b) WNN-GIP, (c) KBMF2K, and (d) WNN-GIP, respectively.
(EPS)

S6 Fig Performance trend of NRLMF on the benchmark datasets (a) Nuclear Receptor, (b) GPCR, (c) Ion Channel, and (d) Enzyme, measured by AUPR with different settings of *K*_2_ under CVS2. The best competitors on these datasets are (a) WNN-GIP, (b) KBMF2K, (c) KBMF2K, and (d) WNN-GIP, respectively.
(EPS)

S7 Fig Performance trend of NRLMF on the benchmark datasets (a) Nuclear Receptor, (b) GPCR, (c) Ion Channel, and (d) Enzyme, measured by AUC with different settings of *K*_2_ under CVS3. The best competitors on these datasets are (a) WNN-GIP, (b) WNN-GIP, (c) WNN-GIP, and (d) WNN-GIP, respectively.
(EPS)

S8 Fig Performance trend of NRLMF on the benchmark datasets (a) Nuclear Receptor, (b) GPCR, (c) Ion Channel, and (d) Enzyme, measured by AUPR with different settings of *K*_2_ under CVS3. The best competitors on these datasets are (a) WNN-GIP, (b) WNN-GIP, (c) BLM-NII, and (d) BLM-NII, respectively.
(EPS)

S9 Fig Performance trend of NRLMF on the benchmark datasets (a) Nuclear Receptor, (b) GPCR, (c) Ion Channel, and (d) Enzyme, measured by AUC with different settings of *c* under CVS2. The best competitors on these datasets are (a) WNN-GIP, (b) WNN-GIP, (c) KBMF2K, and (d) WNN-GIP, respectively.
(EPS)

S10 Fig Performance trend of NRLMF on the benchmark datasets (a) Nuclear Receptor, (b) GPCR, (c) Ion Channel, and (d) Enzyme, measured by AUPR with different settings of *c* under CVS2. The best competitors on these datasets are (a) WNN-GIP, (b) KBMF2K, (c) KBMF2K, and (d) WNN-GIP, respectively.
(EPS)

S11 Fig Performance trend of NRLMF on the benchmark datasets (a) Nuclear Receptor, (b) GPCR, (c) Ion Channel, and (d) Enzyme, measured by AUC with different settings of *c* under CVS3. The best competitors on these datasets are (a) WNN-GIP, (b) WNN-GIP, (c) WNN-GIP, and (d) WNN-GIP, respectively.
(EPS)

S12 Fig Performance trend of NRLMF on the benchmark datasets (a) Nuclear Receptor, (b) GPCR, (c) Ion Channel, and (d) Enzyme, measured by AUPR with different settings of *c* under CVS3. The best competitors on these datasets are (a) WNN-GIP, (b) WNN-GIP, (c) BLM-NII, and (d) BLM-NII, respectively.
(EPS)

S13 Fig Performance trend of NRLMF on the benchmark datasets (a) Nuclear Receptor, (b) GPCR, (c) Ion Channel, and (d) Enzyme, measured by AUC with different settings of *r* under CVS2. The best competitors on these datasets are (a) WNN-GIP, (b) WNN-GIP, (c) KBMF2K, and (d) WNN-GIP, respectively.
(EPS)

S14 Fig Performance trend of NRLMF on the benchmark datasets (a) Nuclear Receptor, (b) GPCR, (c) Ion Channel, and (d) Enzyme, measured by AUPR with different settings of *r* under CVS2. The best competitors on these datasets are (a) WNN-GIP, (b) KBMF2K, (c) KBMF2K, and (d) WNN-GIP, respectively.
(EPS)

S15 Fig Performance trend of NRLMF on the benchmark datasets (a) Nuclear Receptor, (b) GPCR, (c) Ion Channel, and (d) Enzyme, measured by AUC with different settings of *r* under CVS3. The best competitors on these datasets are (a) WNN-GIP, (b) WNN-GIP, (c) WNN-GIP, and (d) WNN-GIP, respectively.
(EPS)

S16 Fig Performance trend of NRLMF on the benchmark datasets (a) Nuclear Receptor, (b) GPCR, (c) Ion Channel, and (d) Enzyme, measured by AUPR with different settings of *r* under CVS3. The best competitors on these datasets are (a) WNN-GIP, (b) WNN-GIP, (c) BLM-NII, and (d) BLM-NII, respectively.
(EPS)

S1 Text The top 1000 novel interactions predicted by NRLMF on the Nuclear Receptor Dataset. (TXT)

S2 Text The top 1000 novel interactions predicted by NRLMF on the GPCR Dataset. (TXT)

S3 Text The top 1000 novel interactions predicted by NRLMF on the Ion Channel Dataset. (TXT)

S4 Text The top 1000 novel interactions predicted by NRLMF on the Enzyme Dataset. (TXT)
